# Application of evidence-based urology in improving quality of care

**DOI:** 10.4103/0970-1591.32055

**Published:** 2007

**Authors:** Arabind Panda, L. N. Dorairajan, Santosh Kumar

**Affiliations:** Department of Urology, Jawaharlal Institute of Postgraduate Medical Education and Research, Pondicherry, India

**Keywords:** Evidence-based urology, quality of care, strength of evidence

## Abstract

Evidence-based medicine requires use of the best available evidence for optimal patient care. Increasingly scarce resources and escalating demands on time have led to emphasis on effective treatment. Opinion is slowly yielding to high-quality existent evidence. It is important that urologists adapt to these changes for them to deliver optimum care to the patients. This article discusses the levels of evidence, the nature of desirable evidence, means of assessing quality of clinical trials and meta-analysis and finally the practice of evidence-based urology with special reference to bedside evidence-based urology.

“It is I and I alone, who have revealed the true path of Medicine.”

**Claudius Galen (AD 129-AD 216)**

Medical progress through the centuries has been based on the teaching of eminent physicians. It was usually based on carefully observed phenomena and backed by the authority of the presenter. Its level of acceptance was based largely on the eminence of the physician and the scientific basis was not vetted to any great extent. So entrenched was eminence-based medicine that it took more than a millennia for Vesalius (1514-1564) to contradict successfully the teachings of Galen.[1] The systematic application of evidence to medical situations first occurred in the realm of epidemiology in the 19^th^ century. Koch stated his postulates and the progress of evidence-based medicine thereafter has been explosive. The randomized controlled trial (RCT) was initially introduced into clinical medicine for the evaluation of the efficacy of streptomycin for tuberculosis in 1948.[2] Further progress in understanding the strength of various study designs has resulted in the RCT being the method of choice to assess the efficacy of interventions. Archie Cochrane, a British epidemiologist initially proposed a case for a more evidence-based approach in the early seventies[3] but it was not until the early nineties that the term was first used in a landmark article by the evidence-based working group.[4] Evidence-based medicine (EBM) as a concept has become firmly established. Around the world, a “trend to evidence” is discernable in healthcare.[5] It utilizes not only RCTs, but all available data.[3] The reasons include increasing calls for accountability of healthcare professionals, the scarcity of resources and preoccupation with cost control and quality. The reductions in working hours in Europe and the USA have necessitated reevaluation of resource and time-intensive procedures in the light of available evidence.[6] This article discusses the levels of evidence, the nature of desirable evidence, means of assessing quantity of clinical trials and meta-analysis and finally the practice of EBM in urology with special reference to bedside evidence-based urology which is of great relevance to the practicing urologist.

## MATERIALS AND METHODS

PubMed and IndMed were searched to identify articles (1985-2006) related to EBM and urology. Key words used included, EBM, evidence-based urology, strength of evidence and quality of care. Recent textbooks on the subject were referred. These were reviewed with focus on application of bedside EBM in urology.

## LEVELS OF EVIDENCE AND GRADES OF RECOMMENDATIONS

EBM is the conscientious, explicit and judicious use of current best evidence in making decisions about the care of individual patients. The practice of EBM means integrating individual clinical expertise with the best available external clinical evidence from systematic research.[7] It is desirable when evaluating a study to place it in a hierarchy of evidence, based on its validity. There exists a multitude of systems developed to categorize studies in their respective levels of evidence. Examples include the Oxford Centre for EBM (OCEBM), American College of Chest Physician etc. The OCEBM system ranks studies of the highest quality (Level 1) to the lowest quality (Level 5)[8] [Table 1]. The different scales have been developed mostly from consensus expert opinion and are not validated. Different systems may categorize the same study into different levels of evidence.

**Table 1:** Levels of evidence

1a	Meta-analysis with homogeneity of randomized controlled trials
1b	Individual RCT (>80% follow-up) with narrow confidence interval
1c	All or none case series*
2a	Meta-analysis with homogeneity of cohort studies
2b	Individual cohort study; low quality randomized controlled trial (<80% follow-up)
2c	Outcomes research; Ecological studies
3a	Meta-analysis with homogeneity of case control studies
3b	Individual case-control study
4	Case series; poor quality cohort and case control studies
5	Expert opinion without explicit critical appraisal or based on physiology, bench research

* *All patients died before the therapy became available, but some now survive on it; or when some patients died before therapy, but none now die on it. Modified with permission from the Oxford centre for evidence-based medicine levels of evidence (May 2001).*[8]

Research studies may be primary, involving clinical research i.e., RCT, cohort, case control studies, case series or case reports. It may be secondary, based on primary data, in the form of a meta-analysis or a clinical practice guideline. A meta-analysis of homogenous RCTs is by general agreement rated as the highest level of evidence.[9] The other end of the spectrum is the expert opinion. In between are the cohort studies, which involve the identification of two groups (cohorts) of patients who received the exposure/treatment of interest and following these cohorts forward for the outcome of interest. The case control study compares cases (a group among whom the problem of interest is present) with controls (a comparison group where the problem is absent) to identify contributing factors. The case series describes a series of patients with an outcome of interest. No control group is present.[10] Levels of evidence from several trials can be tied up to formulate grades of recommendations [Table 2].

**Table 2:** Grades of recommendation

**A**	Consistent level 1 studies
**B**	Consistent level 2 or 3 studies or extrapolations* from level 1 studies
**C**	Level 2 studies or extrapolations from level 2 or 3 studies
**D**	Level 5 evidence or troublingly inconsistent or inconclusive studies of any level

* *Extrapolations are where data is used in a situation which has potentially clinically important differences than the original study situation. Modified with permission from the Oxford centre for evidence-based medicine levels of evidence (May 2001).*[8]

## THE META-ANALYSIS

A meta-analysis evaluates data and results systematically, from different trials on a single subject to arrive at a conclusion about a body of research. It is most frequently applied to RCTs and more recently to observational studies. When methodically sound, it provides a very accurate measurement of the clinical effectiveness of healthcare interventions and precise estimate of treatment effect. The basic requirements of a meta-analysis are well-designed representative trials. Randomized controlled trials based on heterogeneous data offer weaker evidence and if the original studies were poorly designed its conclusion may potentially mislead. In addition, the intervention applied and the settings differ between trials and there may be both loss of information on important outcomes and inappropriate subgroup analysis during a meta-analysis. Moreover, newer experimental data from mega trials on occasion are not always in concordance.[2]

The quality and consistency of reporting of research is varied. Efforts have been made to improve the reporting of RCTs and meta-analysis. These have resulted in the Consolidated Standards for Reporting Trials (CONSORT) guidelines for randomized trials[11] and the Quality of Reports of Meta-analysis of RCT (QUOROM) statement for systematic reviews.[12] Developed by an international group of clinical trialists, statisticians, epidemiologists and biomedical editors they have resulted in improvement in writing, reviewing and assessing trials. Independent groups like the Cochrane collaboration systematically review, summarize and publish databases of relevant topics.[13] It is very difficult for the practicing urologist to be up-to-date about all RCTs relevant to his practice. Hence these systematic reviews and meta-analyses are an important source of evidence.

## EVIDENCE-BASED UROLOGICAL PRACTICE

Systematic reviews/meta-analysis or other primary studies do not provide recommendations or consider all potential consequences of a therapy, i.e., the cost, patient preferences, practicalities. Evidence-based practice systematically acquires, analyses and transfers research findings into clinical, management and policy areas. It involves an amalgamation of evidence, expertise and patient values; blending the science and art of medicine.[4]

The practice of evidence-based urology involves five steps:[14]

### Step 1: Formulating the question

The question has to be framed in a way that can be answered in a systematic review. It must be specific and should include the type of patient, clinical intervention and the outcome of interest.

### Step 2: Stating the criteria for eligibility

The exact inclusion and exclusion criteria must be predecided before searching the literature in order to avoid bias due to arbitrary inclusion and exclusion of certain studies.

### Step 3: Identifying and retrieving relevant articles

A proper literature search to identify articles relevant to the topic of interest is then made to see the best available evidence. Access to the internet and medical database systems greatly facilitate the search. Using different search strategies and keywords can improve results.

### Step 4: Critically appraising the data

Many published articles contain methodical flaws or lack clinical applicability. It is important to determine the quality of the study i.e., the extent to which all aspects of a study design can be shown to protect against systematic bias and inferential error [Table 3].

**Table 3:** Defining outputs

**Experimental event rate (EER)**	The successful outcome rate with the experimental therapy
**Control event rate (CER)**	Rate of the outcome under study in the placebo group
**Experimental event odds**	Ratio of people having the event with the experimental therapy to the number not having the event
**Control event odds**	Ratio of people on placebo, having the event to the number to the number not experiencing the event
**Odds ratio**	A measure of the strength of association between experimental treatment and the outcome. Also known as the cross products ratio, values greater than 1 show the experimental is better than control
**Relative risk and benefit**	The ratio of the EER to the CER. Values greater than 1 show the experimental to be better than control
**Relative risk reduction or Increase (RRR/RRI)**	The difference EER and CER divided by CER. i.e. EER - CER/ CER EER may be subtracted from CER if the experimental event rate is less than the control event rate
**Absolute risk increase or Reduction (ARI/ARR)**	The difference between EER (or CER) and CER (or EER). The effect is solely due to the experimental therapy
**Number needed to treat (NNT)**	It refers to the number of patients necessary to treat to prevent one additional bad outcome. It is the inverse of Absolute Risk Reduction i.e., 1/ ARR.NNT for different therapies can be easily compared and it is an excellent measure of effectiveness.

### Step 5: Applying the evidence to the individual patient

All evidence, however strong is not universally applicable. Clinical judgment is essential when extrapolating evidence to clinical practice.[15,16]

## CLINICAL PRACTICE GUIDELINES (CPG) AND QUALITY OF CARE

The sheer volume of scientific literature makes it rather difficult for even experts to be aware of all relevant literature. A selective skimming of leading journals may result in missed valuable information.[17] A meta-analysis addresses only specific issues and does not address all the issues of therapy. There is therefore a need for systematic reviews and guidelines that consider the entire gamut of management issues.

CPG are not new. While the recommendations of yesteryears were predominantly expert opinion-based, contemporary guidelines are primarily evidence-based and graded according to their strength of evidence. They are an attempt to distill a large body of evidence to a usable format.

Quality of healthcare refers to the degree to which health services for individuals and populations increase the likelihood of desired health outcomes and are consistent with current professional knowledge.[18] Quality improvement depends on evidence-based guidelines, review and audit data. Effective practice of evidence-based urology, along with careful audit of the results may result in improved standards. Clinical practice guidelines in particular, shift practice patterns towards more effective and efficient care. In a recent survey among members of the American Urological Association (AUA), there was widespread agreement that EBM improves quality of patient care and every urologist should be familiar with critical appraisal techniques. Guidelines were used by 91% of respondents reviewed.[19]

## EBM IN UROLOGY AND THE SOCIAL CONTEXT

While guidelines are very useful in evidence-based urological practice they have several limitations. Several assumptions are made during the preparation of a clinical practice guideline. Firstly, a sufficient quantity of valid evidence must exist. Secondly, the majority of healthcare providers will study and implement these guidelines. Third, they will be organized and funded by independent agencies without conflicts of interests. And finally, they should result in an improvement in the quality of care and if possible lead to proper utilization of scarce resources. The process should be methodically sound to produce valid, reliable, clear and applicable recommendations.[20]

Even if these criteria are met, evidence-based recommendations and CPGs need to be sensitive to patient and societal preferences. Guidelines formulated in a particular social and economic context may not find total acceptance in another. For example, the AUA 2003 guidelines for benign hyperplasia of the prostate do not recommend routine ultrasound evaluation (USG) of the upper tract for back pressure changes in patients with benign prostatic hyperplasia.[21] An ultrasound evaluation of the upper tract in the USA will be performed by a radiologist and will cost several hundred dollars; it is therefore economically unviable. However, in India, where USG is relatively less expensive and the same person does upper tract as well as lower tract USG, it is routinely practiced. This practice is all the more relevant in India as many patients present late with chronic retention and deranged renal function because of various social factors in India which are not present in the USA.

**Figure 1:**
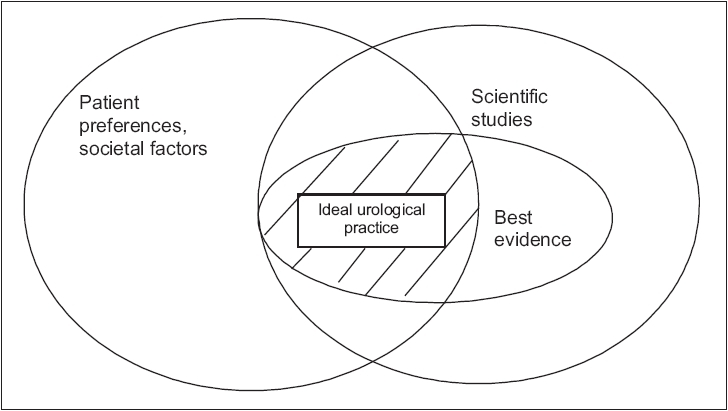
Applying evidence-based urology

Most medical recommendations refer to groups of patients. They may or may not apply to or be acceptable to the patient in question. Sackett *et al* recommend rephrasing the question. “Is my patient so different from those in the trial that the results cannot help me?”[6] The results of a trial should never be used in isolation. It is necessary to consider epidemiology, population needs, social factors and other local priorities. In general when the evidence is relatively weak, e.g., the benefits and harm of routine screening of prostate specific antigen (PSA),[22] patients and providers are likely to give more emphasis to patient values and treatment costs. Conversely, when evidence is strong, patient values may carry less weight in treatment decisions, although their preferences still need to be taken into account [Figure 1].

## BEDSIDE EVIDENCE -BASED UROLOGY

It is not for the research institutions and expert groups alone to evaluate evidence and to make recommendations for other urologists to follow. It is not sufficient for the urologist to say he/she practices EBM by merely following such recommendations. At the individual clinician level EBM is a way of practicing medicine by scientifically evaluating for evidence that the action they take is beneficial to the patient-a method sometimes referred to as “Bedside EBM”. The applicability and relevance of evidence-based medicine in routine patient care shall determine to a great extent its acceptability by urologists. Clinicians can generate around five knowledge “needs” for every inpatient and three “needs” for every outpatient.[23,24] Bedside evidence-based urology is about filling up these gaps in knowledge and its application in a real time setting at the individual level. As described earlier, the needs have to be translated into focused answerable questions, to locate appropriate best evidence and critically appraise the evidence, before application. While certain pre-appraised databases are available, e.g., The Cochrane Database of Systematic Reviews, the vast majority of clinical queries is not reviewed in these libraries, apart from membership issues which might prevent access. In such a case it is essential for the urologist to seek evidence from other sources like textbooks (which are often outdated by the time they are published) or from a PubMed™[25] or IndMed™[26] database for relevant literature. It is essential for the urologist to be able to critically appraise a topic and if possible to record his conclusions, to create a critically appraised topic (CAT) bank.[27] Critically appraised topics so created, are by nature patient- and evidence-based, promoting acquisition of essential literature searching and appraisal skills and its integration with clinical judgment and patient care.

For example in a patient with symptomatic overactive bladder (OAB) is Solifenacin better than Tolterodine? The outcome measures in this case can be-

Its comparable effectiveness in urgency and urge incontinence? Does it have less incidence of dry mouth?

A literature search was made using Pubmed. It took less than 10 minutes to identify an appropriate article for our question. The study by Chapple *et al*, Randomized, double-blind placebo and Tolterodine-controlled trial of the once-daily antimuscarinic agent Solifenacin in patients with symptomatic overactive bladder,[28] deals with these questions and was selected for evaluation of evidence [Table 4].

**Table 4:** Is solifenacin better than tolterodine in overactive bladder?

	Tolterodine 2 mg BD %	Solifenacin 5 mg OD %	Absolute risk reduction %	Number needed to treat
Urgency	62	48	14	7.14
Urge incontinence	42	35	7	4.28
Dry mouth	19	14	5	20

The number needed to treat (NNT) to get one desired outcome in this example was:

Urgency: NNT = 7; possibly the benefits outweigh the risks

Urge incontinence: NNT = 14; the benefits are questionable

Dry mouth: NNT = 20; Difficult to recommend a change for the symptom of dry mouth alone.

A similar analysis of the MTOPS study[29] is published on the Bandolier website to answer the question “Is medical management of benign prostatic hyperplasia (BPH) combination therapy with alpha blockers and 5 alpha reductase inhibitors superior to treatment with a single drug?”[30] The analysis showed that for the prevention of clinical BPH progression in one patient (more than a placebo would) the NNT was 15 for doxazosin, 16 for finasteride and nine for the combination. Similarly, NNT for preventing of one episode of acute retention (more than a placebo would) was not relevant for doxazosin, 60 with finasteride and 52 with combination therapy. The NNT for the prevention of invasive therapy for BPH was not relevant for doxazosin, 31 for finasteride and 27 for the combination therapy. The results show that combination therapy is superior to individual therapy and that alpha blockers don't reduce the chances of acute retention or of invasive therapy. But the relatively high NNT with finasteride or combination therapy (administered over four years) for preventing acute retention and invasive therapy indicates the need to weigh the benefits of avoiding acute urinary retention or invasive therapy with the cost of therapy and the side-effects.

The NNT remains one of the best measures of evidence. It is generally calculated from systematic reviews of RCTs, but it can be quantified from odds ratio and relative risk reduction. The ideal NNT is 1; a favorable outcome occurs in every patient treated and in no patient in the comparison group. The NNTs of very effective treatments are usually in the range of 2-4. The NNTs for prophylaxis will be larger as fewer patients are affected in a large population. For example, the use of aspirin for the prevention of one death after five weeks of myocardial infarction is 40 and yet is regarded as favorable. The NNTs may be negative in the sense that there are more good events with controls than with treatment (e.g., treatment-related side-effects). They are then expressed as the number needed to harm (NNH). Five top English language journals were examined in 1989, 1992, 1995 and in 1998 for how RCTs report their results. Of the 359 articles, eight (2.2%) reported NNTs and 18 (5.0%) the absolute reduction rate.[31]

A CAT is easily made and stored for future reference. It is most valuable as a learning resource if made by the clinician, than obtained secondhand, though these may be available. It may be also generated as a group activity involving multiple residents in an institution or city level urology club and might be more useful than traditional journal clubs.

Individual CATs can be wrong. A CAT is as good as the evidence that went into creating it. Garbage in, garbage out. They also contain a single element of the relevant literature. The article can potentially reflect the bias of the clinician. They also have a short shelf life and need to be periodically updated as newer best evidence is forthcoming. The NNTs too have their drawbacks; they are treatment and period-specific and describe the difference between treatment and control at a particular time. They are best used in trials with comparable outcomes in comparable patients. Where a positive outcome is partly due to a placebo effect, NNT is an overestimate. Also there exists no exact cutoff level for effectiveness.

Self-directed evidence-based learning, critical appraisal and the information generated thereof can improve patient care, confidence in care and potentially affect decision-making in 78% of cases.[32] The Centre for EBM, Oxford has free downloadable software (CAT Maker) that helps to create and store CATs.[8]

## SHORTCOMINGS OF EBM

Urology is not static. New evidence may push previous ideas into obsolescence. Any practice based on previous evidence would have to necessarily give way to new evidence. Unless constantly updated previously appraised topics rapidly lose relevance. Reliable evidence does not exist for many clinical problems, especially the rarer ones. Moreover, randomization of surgical therapy is difficult and may be inappropriate. Any evidence is only as good as the input. Poor quality of the constituent trials will lead to poor quality evidence.

There also is the problem of ‘negative evidence’ that rarely gets published. ‘Publication bias’ and censoring by the authors or their sponsors both contribute. Its lack remains an important impediment in compiling the complete picture. It has been suggested that registering trials with independent agencies and insistence of such registration by journals could reduce the proportion of unpublished evidence.[13,33]

## CONCLUSION

Can urology be evidence-based? Previous fears that EBM may lead to attrition of clinical skills and the art of medicine have been largely unfounded. Most agree on the need, some may quibble on the appropriateness of existing evidence. Practice of evidence-based urology may be a way of keeping abreast of the most relevant developments in the era of exponential growth of urological literature. By helping to hone critical appraisal skills it may possibly improve clinical expertise.

## Appendix: Selected internet resources

1. European Association of Urology http://www.uroweb.org/index.php?structure_id=109
  Evidence-based urology guidelines; free on the internet. E.g.: Male Infertility, Bladder Cancer, Renal Cell Carcinoma, Urinary Incontinence, Urolithiasis
2. American Urological Association http://www.auanet.org/guidelines/
  Evidence-based urology guidelines; free on the internet. E.g.: Erectile Dysfunction, Staghorn Calculi, Premature Ejaculation, BPH, Priapism
3. National Comprehensive Cancer Network http://www.nccn.org/professionals/physician_gls/default.asp
  Clinical practice guidelines in oncology; free on the internet.
  E.g.: Carcinoma bladder, prostate, kidney
4. National Guidelines Clearinghouse http://www.guideline.gov
  Database of clinical practice guidelines, 85 urological guidelines; free on the internet
